# A Group-Based Online Intervention to Prevent Postpartum Depression (Sunnyside): Feasibility Randomized Controlled Trial

**DOI:** 10.2196/10778

**Published:** 2019-05-28

**Authors:** Jennifer Duffecy, Rebecca Grekin, Hannah Hinkel, Nicholas Gallivan, Graham Nelson, Michael W O'Hara

**Affiliations:** 1 Department of Psychiatry College of Medicine University of Illinois at Chicago Chicago, IL United States; 2 Department of Psychological and Brain Sciences University of Iowa Iowa City, IA United States; 3 Serious Mental Illness Treatment Resource and Evaluation Center Department of Psychiatry University of Michigan Ann Arbor, MI United States; 4 Western Carolina University Cullowhee, NC United States; 5 Kansas State University Manhattan, KS United States

**Keywords:** postpartum depression, cognitive behavioral therapy, internet, social support

## Abstract

**Background:**

Postpartum depression (PPD) has a 20% 3-month prevalence rate. The consequences of PPD are significant for the mother, infant, and the family. There is a need for preventive interventions for PPD, which are effective and accessible; however, many barriers exist for women who attempt to access perinatal depression prevention programs. Internet interventions for the treatment and prevention of depression are widely accepted as efficacious and may overcome some of the issues pertaining to access to treatment barriers perinatal women face. However, internet interventions offered without any human support tend to have low adherence but positive outcomes for those who do complete treatment. Internet support groups often have high levels of adherence but minimal data supporting efficacy as a treatment for depression. Taken together, these findings suggest that combining the treatment components of individual interventions with the support provided by an internet support group might create an intervention with the scalability and cost-effectiveness of an individual intervention and the better outcomes typically found in supported interventions.

**Objectives:**

This study aimed to describe the development of a cognitive behavioral therapy (CBT) internet intervention with peer support to prevent PPD and examine preliminary depression and site usage outcomes.

**Methods:**

User-centered design groups were used to develop the internet intervention. Once the intervention was developed, women who were 20 to 28 weeks pregnant with symptoms of depression (Patient Health Questionnaire-8 scores of 5-14) but who had no major depression diagnosis were enrolled in a randomized controlled trial (RCT) to compare 8 weeks of a CBT-based internet intervention with peer support to an individual internet intervention designed to prevent PPD. Assessments took place at baseline, 4 weeks, 8 weeks (end of treatment), and then 4 weeks and 6 weeks postpartum.

**Results:**

A total of 25 women were randomized. Of these, 24 women completed the RCT. Patient Health Questionnaire-9 scores at 6 weeks postpartum remained below the clinical threshold for referral for treatment in both groups, with depression measures showing a decrease in symptoms from baseline to postpartum. At 6 weeks postpartum, only 4% (1/24) met the criteria for PPD. There was no difference between groups in adherence to the intervention, with an average of 14.55 log-ins over the course of treatment.

**Conclusions:**

Results suggest women were responsive to both peer support and individual internet interventions to prevent PPD and that peer support may be a useful feature to keep participants adherent.

**Trial Registration:**

ClinicalTrials.gov NCT02121015; https://clinicaltrials.gov/ct2/show/NCT02121015 (archived by WebCite at http://www.webcitation.org/765a7qBKy)

## Introduction

### Background

Postpartum depression (PPD) has a 20% 3-month prevalence rate [1]. The consequences of PPD are significant for the mother, infant, and the family [2]. Loss of pleasure; low mood; fatigue; difficulty thinking, concentrating, and making decisions; and sleep and appetite disturbance all lead to impairment in daily functioning, especially in caring for an infant. Depressed mothers often show gaze avoidance, more negative and fewer positive facial expressions, and slower or mistimed responses to infant bids for attention [3]. Infants of depressed mothers show less eye gaze during feeding, less play, less positive affect, and higher levels of withdrawal behavior; are more drowsy and fussy; and show higher levels of insecure attachment than infants of nondepressed mothers [2,4]. This negative impact extends to the preschool years and beyond [4,5]. In sum, PPD leads to impairment in maternal behavior, cognition, and affect, which has a clear and negative long-lasting effect on the child. The need for preventive interventions for PPD, which are effective and accessible, is widely recognized.

Many types of face-to-face PPD prevention interventions have been developed and tested. Cognitive behavioral therapy (CBT), interpersonal psychotherapy, and antidepressant medications have demonstrated benefit in preventing the onset of depressive symptoms [6,7]. CBT has considerable support in preventing major depressive episodes as well perinatal depression. CBT focuses on teaching patients to identify and challenge dysfunctional beliefs and problematic behaviors. It has been shown to be effective in numerous settings, including individual-, group-, and distance-based interventions [8]. However, many barriers exist for women who attempt to access traditional perinatal depression prevention or treatment programs. Barriers may exist on multiple levels (patient, provider, and practice) [9] and make it difficult for women to access services, even if they have been identified as having depressive symptoms or being at risk for developing them. Barriers include stigma, cost, scheduling difficulties, and lack of providers and programs [10-12]. Structural barriers such as time constraints, lack of child care, and transportation also are substantial barriers to obtaining psychological treatment among 75% of depressed urban primary care patients [13]. Online prevention and intervention programs have the potential to overcome many of these barriers.

Internet interventions for the treatment of depression are widely accepted as efficacious [14-18]. Though prevention interventions are fewer, they have demonstrated support [19-21]. These interventions commonly consist of didactic material and interactive tools to practice skills, such as cognitive restructuring or behavioral activation. Stand-alone individual interventions (those without any human support) typically show smaller effect sizes than coach-supported interventions, likely because of decreased adherence. Although coach-supported interventions may have improved outcomes, drawbacks include increased cost and decreased scalability. Internet support groups (ISGs), where peers provide support rather than trained coaches, are frequently utilized by pregnant women and new mothers, with 75% of mothers endorsing use of internet-delivered support [22]. However, despite good adherence and high levels of interest, ISGs have limited data supporting their efficacy for treating or preventing depression [23-26].

### Objectives

Taken together, these findings suggest that combining the treatment components of individual interventions with the support provided by ISGs may create an intervention with the scalability and cost-effectiveness of an individual intervention and the outcomes similar to those found in coach-supported interventions. There have been a number of small trials that suggest that peer support has the potential to improve adherence and depressive outcomes [23,27,28], and this study sought to examine the use of this approach in the perinatal population. The aims of this study are to describe the development of a CBT internet intervention with peer support to prevent PPD and examine preliminary depression and site usage outcomes.

## Methods

### Stage I: User Centered Design Groups

#### User-Centered Design Groups

With institutional review board approval, user-centered design groups were conducted to engage women from the target population in the intervention-building process (topics, site motif, and usability of potential application). User-centered design groups were held from July to September 2014, with a total of 6 participants across 3 groups. A total of 2 groups were held in-person on campus at 1 of the participating institutions; the third group was held online for ease of data collection and participation. All were facilitated and analyzed by a psychologist consultant for this grant and analyzed using a phenomenological approach to qualitative analysis [29]. No software was used.

#### Participants and Procedures

Participants were recruited by word-of-mouth from investigators at the 2 participating institutions and through recruitment flyers distributed around the campus of 1 institution. Participants ranged in age from 25 to 45 years, reported median income of US $100,000.00 and education level as graduate or postgraduate. Furthermore, 100% of the sample identified as white, and 100% were partnered or married. Of them, 1 participant was still pregnant (0 children), 2 participants reported having 2 children each, and 3 participants reported 1 child in their household.

Before beginning the groups, a moderator guide was developed to ensure consistency of questions and topics covered. Low-fidelity prototypes were used to obtain feedback on various site components and motifs. All groups were shown the same set of materials. Questions were formulated along 3 lines of inquiry: (1) pregnancy topics of interest (topics about which women might be seeking more information); (2) motifs for the intervention (visual themes and look and feel of the internet site); and (3) use of the intervention (how, when, and why they might interact with the intervention).

Investigators defined pregnancy topics of interest in initial meetings regarding the creation of the intervention and vetted these with group participants. The initial list was developed by consulting the literature and brainstorming among the authors and collaborators, who have extensive clinical experience with pregnant women. Investigators also asked for any topics participants did not see included in the premade list. Participants were initially asked for motifs they had either seen or thought of themselves in response to pregnancy, mothering, babies, etc. In total, 3 motifs for the intervention had already been created by the research team in conjunction with the application development team and were vetted among group participants. Finally, participant interest in using the proposed intervention was gauged using diagrams of predetermined themes, potential topics as discussed in the focus group, and the broader concept of social media for information and community. Predetermined motifs were developed through brainstorming sessions with the authors, program designers, and graphic designers.

### Stage II: Pilot Trial

#### Study Design

Participants were randomized in groups of 7 to 9 to either the Share (group) condition or the Control (individual) condition. In total, the Share condition comprised 18 women, and the Control condition comprised 7 women. Randomization of 2:1 was utilized to gain more experience with the group intervention component, which was more novel than the individual intervention. Contact was lost with 1 participant in the Share condition immediately after randomization, so 17 women actually participated in the Share condition. Subsequent to randomization, participants had an initial 20-min phone call with the study staff to establish rapport, ensure site functionality, and elicit change-talk via motivational interviewing. Study staff had no additional contact with participants except for regularly scheduled assessments. Study data were collected and managed using REDCap electronic data capture tools hosted at the University of Iowa (UI) [30].

#### Website

The Sunnyside website was an 8-week online prevention intervention developed by research partners at the Northwestern University [31]. The Sunnyside website is based on cognitive behavioral principles [32] that consisted of 16 core didactic lessons (plus 3 postpartum booster sessions) and 5 associated tools. See Multimedia Appendix 1 for an overview of intervention content. CBT was selected as the intervention approach given the evidence base supporting its effectiveness in managing perinatal depression as well as the authors’ experience in creating online CBT interventions [31,33]. Each lesson, which required 10-15 min to complete, was uniquely designed to provide information about pregnancy and postpartum issues, as well as the components of CBT. At the conclusion of each lesson, women were prompted with a *Call to Action* slide that encouraged them to directly apply the CBT strategies that were learned in the lessons. The lessons comprised text and video material. In addition to the 8 weeks that comprised the core portion of the intervention, participants also completed postpartum booster sessions, which were lessons made available at 2 weeks, 4 weeks, and 6 weeks postpartum.

New lessons and accompanying tools were released twice a week, and participants received an email notification upon the release of this material. The initial lesson introduced the cognitive behavioral principles utilized throughout the intervention and explained how one’s thoughts and behaviors affect their moods and physical being. The program contained 5 interactive cognitive behavioral tools: thought restructuring (*Think*), mood tracking (*Feel*), activity scheduling and monitoring (*Do*), relaxation (*Relax*), and goal setting (*Achieve*). Associated tools served to complement the lessons by having the women directly apply the CBT strategies that were discussed in the lessons. See Multimedia Appendix 1 for more information on the content of the lessons and tools. Taken together, the lessons and tools provided useful information and additional resources on how to manage mood and cope with depression and anxiety.

#### Experimental Intervention: Share

Content and general layout was identical for the Control and Share, with the exception that the Share site featured a newsfeed and accountability features. Although the Control participants focused on the lessons and interactive tools on the website, the Share participants also collaborated through the *Activity Feed*. The *Activity Feed* was a constantly updating feed that displayed each of the women’s activity on the site. Here, participants were able to post, *like*, and comment or provide feedback to other women’s posts. Discussion questions were posted with the release of each lesson to encourage interaction.

Share participants also maintained an *Individual Garden Plot* as well as a *Community Garden* that were linked to user profiles. Women provided information about themselves in the profiles to increase group bond. In both of the garden plots, incentives, such as garden gnomes or flower collections, were earned by completing various tasks on the site, such as reading a new lesson or posting on the feed, but they were only added to the Community Garden once each of the group members had completed the identified task. The flower garden provided a visual representation of each participant’s site use to increase accountability to each other on task completion. Women were also able to reach out to each other with a generic *nudge* message that sent an email indicating that a specific group member requested return to the site. A deliberate decision was made to not allow private messaging between participants to encourage interaction on the site.

Women were also provided a *Contact Moderator* tool to report any issues that arose with either the site or with group members. Staff members watched over the site to verify that medically inaccurate information was not being posted and to ensure this was a safe space for participants to disclose their feelings.

#### Measures

Participants completed a total of 5 study assessments, which included self-reported online questionnaires and interview-based assessments that were conducted over the phone by trained graduate students in clinical psychology. Outcomes were assessed at baseline, week 4 of the treatment program, week 8 (end of treatment), 4 weeks postpartum, and 6 weeks postpartum. Interview assessments were conducted at baseline, week 8, and 6 weeks postpartum. Self-report measures were collected at all 5 time points. Assessors were blinded to site arm and intervention usage. Participants were compensated US $20 per assessment completed, for a total of US $100 over the course of the study. Compensation was tied to assessment completion, not site usage, to decrease loss to follow-up in assessment even if participants were no longer engaged with the site.

#### Use

Use was examined through total number of log-ins, completion of tools, and lessons. Peer support features (likes, comments, nudges, and posts) were examined for those in the Share condition.

#### Usability and Satisfaction

Usability and satisfaction were measured using the 17-item Usability, Satisfaction and Ease of Use questionnaire (USE) [34] which was designed to measure satisfaction, usefulness, ease of use, and ease of learning on a 1-7 Likert scale, with higher numbers indicating greater usability and satisfaction.

#### Depressive Symptoms

The Hamilton Depression Rating Scale (HDRS) is a 17-item scale that assesses the severity of depression symptoms. Participants are scored on a range of severity (0-4) or incidence (0-2), based on the variable. Variables include depressed mood, agitation, and somatic symptoms, among others [35]. Data on the structured HDRS support interrater reliability, internal consistency, and high test-retest reliability [36].

The Inventory of Depression and Anxiety Symptoms (IDAS) is a self-report tool that aims to assess specific dimensions of depression and anxiety symptomatology. It contains 10 symptom-specific scales, including suicidality, appetite, and panic, among others, as well as 2 broader scales for general depression and dysphoria. This inventory possesses both internal consistency and content validity [37]. The 20-item general depression scale was used for this study.

The Patient Health Questionnaire (PHQ-8) is an 8-item modification of the Primary Care Evaluation of Mental Disorders used to provide diagnostic criteria for depression symptoms and commonly used in depression screening. The Patient Health Questionnaire-9 (PHQ-9) is a replica of the 8-item form with the addition of a suicidality item. Participants are scored based on the frequency of certain moods and behaviors over the last 2 weeks, from 0 (not at all) to 3 (nearly every day) [38]. The internal reliability and test-retest reliability of this measure was excellent with a Cronbach alpha of .89 and .84, respectively, and correlates strongly with other mental health assessments [39]. The PHQ-8 was administered during the initial online screening, whereas the PHQ-9 was used for all other assessments.

#### Psychiatric Diagnosis

The Structured Clinical Interview for Diagnostic and Statistical Manual of Mental Disorders (DSM) Axis-I Disorders (SCID-I) is a semistructured interview that guides the diagnosis of the major DSM Axis-I disorders. Its modules show excellent to good reliability and superior validity compared with clinical interviews (First, 1995). Suicidality was assessed using the suicide question from the Mini International Neuropsychiatric Interview (MINI), a brief structured interview for the diagnosis of DSM and International Classification of Diseases disorders [40].

#### Statistical Analyses

As this was a pilot study, descriptive statistics were used to examine the data. Results should be interpreted with caution because of the small sample size.

#### Procedures

##### Eligibility

Eligible participants were 18 years of age or older, between 20- and 28-weeks gestation at the time of baseline assessment, had a score between 5 and 14 on the PHQ-8 screener (mild-moderate depressive symptoms), were able to read and speak English, and had access to the internet on any device. Exclusion criteria included diagnosis of a major depressive episode, psychotic disorder, bipolar disorder, substance use disorder or other diagnoses using the MINI, current use of psychotropic medications, intention to resume antidepressant medication after delivery (if women discontinued use during pregnancy), currently in psychotherapy, and endorsed suicidality (with separate procedures in place for responding to these women). The rationale for the PHQ-8 eligibility criteria is that subthreshold depression symptoms are a common criterion for entry to prevention programs [41,42]. The purpose of prevention interventions is to halt the progress toward PPD; thus, identifying those already on the trajectory is important.

##### Recruitment

All women who met eligibility criteria from May 2015 to August 2015 were invited to participate in the study. Participants were identified by the UI’s Institute for Clinical and Translational Science (ICTS). The ICTS accessed the medical records from the UI Hospitals and Clinics and generated a list of women who were currently pregnant and 18 years of age and older. UI research team members followed up with the women generated from that list to inquire about interest in participation. In addition, UI and University of Illinois-Chicago employed use of mass-email and advertisement via Research Match.

Interested participants were directed to the study Web page where they completed the initial online screener. Consent was obtained before completion of the screening questionnaire. Eligible women were invited to complete the baseline clinical phone interview and self-report assessments, which were the final step in determining eligibility for the study. Once women completed the assessments and were deemed eligible, they were randomized into a cohort. This is discussed in greater detail below.

All procedures were approved by the institutional review boards at the UI, Northwestern University, and University of Illinois-Chicago.

## Results

### Stage I: User Centered Design Groups

#### Topics

Topics to be included in the final version of the intervention fell within 3 common categories—physical changes (mother and fetus), logistical challenges, and emotional stresses.

In terms of physical changes, 50% (3/6) of women reported that including information on body changes—such as weight, body after baby, changes during and after pregnancy, and breastfeeding—would be helpful. Participants suggested that simple psychoeducation itself would ease anxiety levels but they had difficulty finding useful information. Logistical challenges—including the broad *different strategies work for different kids* and the specific *When to amend your will* were the second most common responses in terms of topic category. Finally, 100% (6/6) of women described some sort of emotional challenge they had anticipated at some time during their pregnancy, including the fears of *Am I meant to be a mother* and *the experience of having a baby is not “easy” or “happy” or “blissful.”* Each woman described including these topics in an app designed to prevent PPD or at least recognize it early on if it were to occur, as a helpful, if not essential, goal.

#### Motifs

In reviewing predetermined themes offered for the overall appearance of the app, the overwhelming favorite (6/6, 100%) was a flower garden. This theme seemed to reflect the undercurrent of *new* and *emerging* in spontaneous participant answers. In total, 1 participant stated:

...the flower theme...I think it has a positive tone of growing and new life.

Participants also felt strongly and negatively toward the predetermined themes of Egg in a Nest and Fish Bowl. Of them, 1 participant commented:

Not sure about the eggs...reminds me of something to eat.

The same participant more strongly added:

I don’t know what I would want—but I know I wouldn’t want anything with teddy bears, ducks, or other baby animals.

#### Use

Participants were asked a number of questions about their anticipated or hypothetical use of an intervention during pregnancy. Participants suggested notifying program participants of new content via any number of messaging systems, including text, email, push notification, or pop-up within the app. Participants suggested the information in the intervention should be credible, monitored by an authority or third-party figure, and should include information about how to use the app, as well as how to be social on the app. In this way, study participants could create a supportive, informative community. Finally, participants suggested incentivizing social interaction on the intervention with changes in the flower garden environment, such as growth or more flowers in the garden as interpersonal activity or use of the app increases.

### Stage II: Pilot Trial

#### Participants

**Figure 1:**
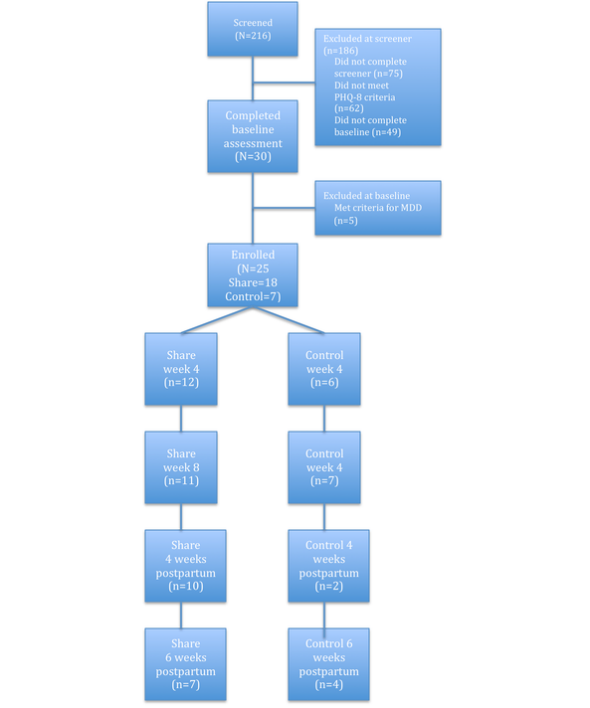
CONSORT flow diagram. MDD: major depressive disorder; PHQ-8: Patient Health Questionnaire-8.

A total of 24 pregnant women (1 additional woman ceased participation immediately after randomization) in their second trimester participated in exchange for compensation. The sample was predominantly white (17/24, 72%), with 8% (2/24) African American, 8% (2/24) Asian or Asian American, 8% (2/24) multiracial, and 4% (1/24) Latina. The average age was 30.5 years (SD 4.05). The majority of the sample was married or cohabitating (20/24, 83%) and were employed either part-time or full-time (16/24, 67%). PHQ-8 scores at screening were 6.7 (SD 1.6). In total, 216 women completed the online screener. Most women screened out during the online screening process (n=180). Of those who completed the baseline assessment, 83% (*n*=25) were eligible and enrolled in the trial, which is a similar rate to other online depression prevention trials [21]. A total of 5 women (17%) were excluded because of current major depressive disorder (MDD) symptoms. See Figure 1 for more information.

#### Attrition and Site Use

Overall, 1 participant in the Share group withdrew from participation before beginning the intervention. Mean number of log-ins across the 8-week intervention plus booster sessions was 12.6 (SD 6.9) for Control (N*=* 7) and 16.5 (SD 9.3) for Share (N*=* 17). The average number of lessons accessed (Control: mean 12.1, SD 5.6; Share: mean 13.7, SD 4.3) and tools utilized (Control: mean 31.0, SD 28.6; Share: mean 24.3, SD 17.6) was similar between groups. Data are provided in Table 1.

Use of peer support features was fairly low (mean 5.9, SD 5.6); however, 59% (10/17) of the participants used at least one feature. Commenting on discussion questions or posts was most popular (59%); initiating status updates also was common (53%). The *like* and *nudge* features were utilized by fewer participants (35% and 12%, respectively).

**Table 1:** Adherence data.

Program activity	Control (N=7)		Share (N=7)	
	Mean (SD)	Range	Mean (SD)	Range
Log-ins	12.6 (6.9)	5-21	16.5 (9.3)	2-35
Lessons accessed	12.1 (5.6)	2-17	13.7 (4.3)	2-18
Tool: activity scheduling/monitoring	10.7 (15.6)	1-34	6.2 (4.1)	1-12
Tool: mood rating	15.8 (9.9)	5-30	12.4 (9.5)	1-33
Tool: relaxation	7.7 (2.5)	5-10	3.4 (1.8)	1-6
Tool: thought record	6.5 (3.8)	1-11	5.1 (2.8)	1-11
Tool: goal setting	3.0 (0.0)	3-3	2.7 (3.1)	1-11
Total tools used	31.0 (28.6)	1-81	24.3 (17.6)	5-63

#### Usability and Acceptability

At week 8, scores on USE subscales ranged from 1 (strongly disagree) to 7 (strongly agree) and fell in the average range for both groups. Control participants’ mean scores were 3.69 (SD 1.76) for usefulness, 4.94 (SD 0.88) for ease of use, 5.77 (SD 0.91) for ease of learning, and 3.93 (SD 1.70) for satisfaction. Share participants’ mean scores were 4.42 (SD 0.87) for usefulness, 4.97 (SD 0.74) for ease of use, 5.73 (SD 0.87) for ease of learning, and 4.58 (SD 1.11) for satisfaction.

#### Depression

PHQ-9 scores at 6 weeks postpartum remained below 10, the clinical threshold for referral for treatment in both groups [39], with measures showing a decrease in symptoms from baseline to postpartum with the exception of the PHQ-9. PHQ-9 scores in the Control group increased slightly from baseline to 6 weeks postpartum, but still remained below a clinically significant threshold. Of them, 1 woman in the Control group met the criteria for a major depressive episode at 6 weeks postpartum diagnosed via the SCID. See Table 2 for more information.

**Table 2:** Depression outcomes.

Outcome measures		Baseline	4 weeks	8 weeks	4 weeks postpartum	6 weeks postpartum	Difference
**Control, mean (SD)**							
	PHQ-9^a^	5.6 (3.5)	4.4 (3.7)	7.3 (4.8)	3.0 (0)	7.2 (5.4)	+1.6 (1.9)
	HDRS^b^	8.3 (2.9)	—^c^	6.2 (3.9)	—	5.5 (4.0)	−2.8 (1.1)
	IDAS^d^	47.3 (7.8)	42.4 (9.8)	41.6 (13.7)	45.0 (7.1)	41.3 (9.0)	−6.0 (1.2)
	SCID^e^	0	—	0	—	1	—
**Share, mean (SD)**							
	PHQ-9	5.1 (3.6)	5.8 (4.3)	3.7 (2.3)	2.3 (1.3)	3.7 (3.8)	−1.4 (.2)
	HDRS	8.6 (5.4)	—	3.6 (2.0)	—	3.3 (2.3)	−5.3 (3.1)
	IDAS	45.4 (6.3)	43.9 (6.6)	37.7 (4.7)	36.9 (7.0)	36.6 (4.8)	−8.8 (1.5)
	SCID	0	—	0	—	0	—

^a^PHQ-9: Patient Health Questionnaire-9.

^b^HDRS: Hamilton Depression Rating Scale.

^c^Not applicable.

^d^IDAS: Inventory of Depression and Anxiety Symptoms.

^e^SCID: Structured Clinical Interview for DSM Disorders.

## Discussion

### Principal Findings

This study outlines the development and initial testing of a novel Web-based intervention to prevent PPD. It indicates that pregnant women are willing to use an individual intervention or a group-based program and that doing so may impact the development of depressive symptoms. At the completion of the trial, only 1 woman (5%) in this at-risk sample met criteria for PPD compared with a 17% prevalence rate seen in at-risk women in the absence of an intervention [43]. Depressive symptoms decreased across most measures from baseline to postpartum in both the Share group and the Control individual condition. Given the adverse effects of depression on both mothers and babies, the reduction in symptoms is positive.

Intervention use was high as compared with other online PPD prevention interventions, with no significant difference between groups. For example, for pregnant women in the Mamma Mia trial, an automated Web-based PPD prevention intervention, the average number of sessions was 7.4 (of 16) [19]. The e-MB trial, another Web-based individual intervention for PPD prevention, found reasonable adherence (40.47 min of intervention use) but did not have a significant impact in the development of PPD [20]. In the MMB program, an online individual intervention utilizing a mindfulness-based cognitive therapy approach, 57% of women completed at least 4 out of 8 sessions, with the average number of sessions completed at 4.72 [21]. As discussed in the development of the NetMums online PPD treatment intervention, perinatal women have specific needs that are often overlooked in Web-based CBT programs focusing on MDD [44]. Content in Sunnyside was carefully designed to provide desired pregnancy information as well as mood management material, and it is possible that this helped draw women back to the site.

Usability scores indicate that the overall user experience was satisfactory. However, limited uptake of the peer network features suggest that changes could be made to increase group discussion and cohesion. Increased utilization of peer support features might lead to greater differences between groups but given that both groups showed adequate adherence and positive outcomes, optimization of those features may not provide any further benefit.

Although dropout from assessments was high from baseline to 6 weeks postpartum, we retained 72% of participants in assessments through the completion of the intervention. Retention at follow-up dropped considerably, in part because of the time demands on the new mothers. In future trials, this problem will have to be addressed by requiring less time for assessment and doing a better job of preparing participants for the postpartum assessments.

### Strengths and Limitations

There are several strengths in this trial. To our knowledge, this is the first online intervention for PPD prevention that included peer social support. Social support is consistently found to be beneficial for pregnant women but can be difficult to access for many women. Women consistently utilized the intervention, even without human support, suggesting that this modality of intervention is appealing to pregnant women.

The limitations of this trial include small sample size, limited diversity in the sample, and the short duration of follow-up. Ideally, follow-up would be closer to 6 months or 1 year to determine if PPD developed later in the postpartum period. Results should be interpreted with caution because of the small sample size, and a larger trial with a more diverse group is needed to verify the outcomes. In addition, most of our participants were recruited via electronic methods, such as email or Research Match, the online volunteer registry. This suggests participants were comfortable using technology. The results may not generalize to those without familiarity with the internet.

### Conclusions

In conclusion, this study outlines the development process and feasibility testing of a Web-based intervention to prevent PPD. Results suggest that women were responsive to the intervention although it would benefit from continued refinement. Next steps include a larger trial with a more diverse sample and a longer follow-up period. In addition, making intervention enrollment available via primary care clinics or ob-gyn offices, rather than simply online, may increase access for those most in need of preventive services but with less familiarity with online resources.

## Appendix

Multimedia Appendix 1 Intervention description.

Multimedia Appendix 2 CONSORT-eHealth Checklist (V 1.6.1).

## Abbreviations

CBT: cognitive behavioral therapy
DSM: Diagnostic and Statistical Manual of Mental Disorders
HDRS: Hamilton Depression Rating Scale
ICTS: Institute for Clinical and Translational Science
IDAS: Inventory of Depression and Anxiety Symptoms
ISGs: internet support groups
MDD: major depressive disorder
MINI: Mini International Neuropsychiatric Interview
NIH: National Institutes of Health
PHQ-8: Patient Health Questionnaire-8
PHQ-9: Patient Health Questionnaire-9
PPD: postpartum depression
RCT: randomized controlled trial
SCID-I: Structured Clinical Interview for DSM Axis-I Disorders
UI: University of Iowa
