# Supplementary material for: A Group-Based Online Intervention to Prevent Postpartum Depression (Sunnyside): Feasibility Randomized Controlled Trial
Source: JMIR Ment Health. 2019 May 28;6(5):e10778. doi: 10.2196/10778 (PMC6707575; doi:10.2196/10778)
Supplement: Multimedia Appendix 1 [file mental_v6i5e10778_app1.pdf]

Table 1. Intervention description.

Week	Lesson content	Associated tool
Week 1		
Part 1	**Your Mood and Your Pregnancy**: An introduction to the principles and risk factors of depression during pregnancy	**The Feel Tool**: Participants are encouraged to rate their mood each time they visit the site to get a better sense of their day-to-day feelings
Part 2	**Worries about Me and my Baby**: Articulated worries that mothers-to-be have during their pregnancy and provides helpful insights into those worries	Participants are reminded to keep using the **Feel Tool**
Week 2		
Part 1	**Mood Management**: An introduction to the CBT principles of the program and how one's thoughts and behaviors affect their moods and physical being	**The Think Tool**: Used to track one's thoughts and discern between helpful and harmful thinking
Part 2	**Challenging Your Thinking**: Participants are provided with strategies to help combat their negative thought patterns and learn new adaptive thoughts	Participants are reminded to keep using the **Think Tool**
Week 3		

Part 1	**Positive Activity During Pregnancy**: Described how their behaviors can affect their mood and the importance of planning positive activities	**The Do Tool**: Participants track their daily behaviors, identify any patterns in their actions, and plan future positive activities
Part 2	**Physical Activity During Pregnancy**: Participants are reminded of the importance of physical activity during pregnancy and are provided with pregnancy-related exercise tips	Participants are requested to use the **Do Tool** to plan physical activities as well as the **Feel Tool** to track how those activities impact their mood
Week 4		
Part 1	**Partner Communication and Support**: The importance of communication and how to keep partners involved in pregnancy and postpartum	Participants are encouraged to make a commitment with their partner via the **Do Tool**
Part 2	**Body Image and Sex During Pregnancy and the Postpartum**: A reminder of the importance of nutrition during pregnancy and facts about sex and intimacy in pregnancy and postpartum	Participants are encouraged to use the **Think Tool** to work through negative thoughts about sex and pregnancy
Week 5		

Part 1	**Relationships with your Mother and Mother-in-Law**: Maintaining healthy relationships with and expectations of her mother and mother-in-law in pregnancy is discussed	Participants are encouraged to use the **Do Tool** to plan activities with their mother or mother-in-law and are introduced to the **Relax Tool**
Part 2	**Challenges in Relationships with Friends and others**: Participants are reminded of the importance of maintaining relationships with friends and family, asking for help when needed, and to setting boundaries to avoid overwhelming	Participants are encouraged to reach out to friends and use the **Think Tool** if any worries arise
Week 6		
Part 1	**Monitoring Kick Counts and Other Pregnancy Anxiety**: Feelings of uncertainty during pregnancy are discussed, including breastfeeding, baby's kicking, and the formulation of a birth plan	**The Relax Tool**: Relaxation and mindfulness strategies are examined to help manage anxiety and mood
Part 2	**Anxiety and Parenthood**: Support systems for worries about parenthood are discussed, as well as an introduction to the Mind-Body Connection	Participants are encouraged to keep using the **Relax Tool**
Week 7		

Part 1	**Relaxation**: The Mind-Body Connection is further discussed, and participants are introduced to specific relation and mindfulness techniques	Participants are encouraged to keep exploring the instruments of the **Relax Tool**
Part 2	**Employment Issues**: Participants are trained how to cope with pregnancy and postpartum symptoms at work, and maternal leave is discussed	Participants are reminded to use the **Do Tool**
Week 8		
Part 1	**During and After the Birth: How to Manage and Resources**: A review of the stages of labor, pain management during childbirth, and postdelivery tips for when the women are home with their newborns	Participants are encouraged to use the **Think Tool** to help process their thoughts about childbirth
Part 2	**Moving Forward and Conclusions**: Participants are given a final summary of the cognitive behavioral lessons they have learned and are reminded of the signs and symptoms of *the blues* and PPD	No specific Tool associated with this lesson
Booster		

Lesson 1	**Welcome Back**: Previously discussed topics are revisited, including PPD and *the blues*, asking for help, and setting boundaries	Participants are reminded to use the **Think Tool**
Lesson 2	**Relationships and Unhelpful Thoughts**: Participants are reminded of the importance of their bond with their partner, and the cognitive behavioral principles of the program were revisited	No specific Tool associated with this Lesson
Lesson 3	**Tracking and Challenging Thoughts and Planning Pleasant Activities**: The connection between thoughts and positive activities are reexamined, and relaxation and mindfulness strategies are reviewed	No specific Tool associated with this Lesson
